# Targeting *S. aureus* Extracellular Vesicles: A New Putative Strategy to Counteract Their Pathogenic Potential

**DOI:** 10.3390/pharmaceutics16060789

**Published:** 2024-06-11

**Authors:** Giulio Petronio Petronio, Maria Di Naro, Noemi Venditti, Antonio Guarnieri, Marco Alfio Cutuli, Irene Magnifico, Alessandro Medoro, Emanuele Foderà, Daniela Passarella, Daria Nicolosi, Roberto Di Marco

**Affiliations:** 1 Department of Medicina e Scienze della Salute “V. Tiberio”, Università degli Studi del Molise, 86100 Campobasso, Italyroberto.dimarco@unimol.it (R.D.M.); 2 Department of Drug and Health Sciences, Università degli Studi di Catania, 95125 Catania, Italy; 3 UO Laboratorio Analisi, Responsible Research Hospital, 86100 Campobasso, Italy; 4 Aileens Pharma S.r.l., 20834 Nova Milanese (MB), Italy

**Keywords:** atopic dermatitis, *Staphylococcus aureus*, gram+ bacterial extracellular vesicles, *Cutibacterium acnes*, skin barrier function, tight junctions, keratinocyte cell death

## Abstract

Long-term inflammatory skin disease atopic dermatitis is characterized by dry skin, itching, and eczematous lesions. During inflammation skin barrier protein impairment promotes *S. aureus* colonisation in the inflamed skin, worsening AD patient’s clinical condition. Proteomic analysis revealed the presence of several immune evasion proteins and virulence factors in *S. aureus* extracellular vesicles (EVs), suggesting a possible role for these proteins in the pathophysiology of atopic dermatitis. The objective of this study is to assess the efficacy of a wall fragment obtained from a patented strain of *C. acnes* DSM28251 (c40) and its combination with a mucopolysaccharide carrier (HAc40) in counteract the pathogenic potential of EVs produced by *S. aureus* ATCC 14458. Results obtained from in vitro studies on HaCaT keratinocyte cells showed that HAc40 and c40 treatment significantly altered the size and pathogenicity of *S. aureus* EVs. Specifically, EVs grew larger, potentially reducing their ability to interact with the target cells and decreasing cytotoxicity. Additionally, the overexpression of the tight junctions mRNA zona occludens 1 (ZO1) and claudin 1 (CLDN1) following EVs exposure was decreased by HAc40 and c40 treatment, indicating a protective effect on the epidermal barrier’s function. These findings demonstrate how Hac40 and c40 may mitigate the harmful effects of *S. aureus* EVs. Further investigation is needed to elucidate the exact mechanisms underlying this interaction and explore the potential clinical utility of c40 and its mucopolysaccharide carrier conjugate HAc40 in managing atopic dermatitis.

## 1. Introduction

Atopic dermatitis (AD) is a chronic inflammatory skin disease characterized by eczematous lesions with pruritus and xerosis. AD skin lesions have specific features, including disrupted barrier function with epidermal hyperplasia and *Staphylococcus aureus* colonization [1,2,3]. A deficit of physiological skin barrier functions caused by keratinocyte death is considered to be the major etiopathogenetic early triggering factor in AD. Pathogen-associated antigens and allergens can enter the skin through these compromised barriers, affecting the host immune responses. The skin lesions of AD patients show increased keratinocyte death triggered by immune mediators. *S. aureus*, an opportunistic pathogen, releases virulence factors like α-toxin, δ-toxin, and exfoliative toxins, contributing to skin pathologies [4,5,6,7]. It is closely related to AD because it colonizes the skin lesions of most AD patients and augments disease severity [8]. *S. aureus* affects the host immune system by producing harmful molecules and toxins, such as staphylococcal enterotoxins and hemolysins [9]. Among these toxins, α-hemolysin (also called α-toxin) is particularly important as it targets keratinocytes and is associated with the severity of AD [8]. Recent findings suggest that *S. aureus* secretes extracellular vesicles (EVs) containing a variety of proteins, DNA, RNA, and toxins, thereby contributing to the pathogenesis of atopic dermatitis (AD) [10]. Remarkably, research conducted by Hong et al. and Jun et al. [8,11] demonstrated the pivotal role of α-hemolysin, a component of EVs, in triggering AD-like skin inflammation. This effect was linked to the disruption of the skin barrier through keratinocyte necrosis and an increased synthesis of pro-inflammatory mediators by keratinocytes. Additionally, it was observed that *S. aureus* colonization on the skin of AD patients resulted in the secretion of α-hemolysin, and its levels were closely associated with the severity of AD. Therefore, α-hemolysin, particularly in its EV-associated form, emerges as a potential target for the diagnosis and treatment of AD. EVs derived from *S. aureus* have been found to contain pathogenic toxins, including α-hemolysin [10], with β-hemolysin also implicated in some studies and correlated with AD.

The skin’s primary physiological function involves providing a physical barrier against external environmental factors. This barrier is predominantly maintained by tight junctions (TJs), which play a crucial regulatory features throughout skin aging, responsible for maintaining the epidermal barrier’s integrity and balance. These TJs provide an important role in maintaining the skin barrier’s strength and functionality by carefully regulating the flow of water and substances through the skin’s outer layers. It has been discovered that the proteins in TJs decrease in aging skin areas, influencing a variety of skin health factors [12,13].

These structures regulate not just the transport of chemicals through the skin, but also the transit of immune cells, helping the skin defend itself against the infiltration of pathogens, diseases and toxic substances [13,14,15]. These key functions are integrally engaged in the aging process [16].

Several TJ proteins with diverse epidermal localization patterns have been identified, including occludin, cingulin, zonula occludens (ZO)-1, and claudins [14,17]. The colonizing microbiota also critically modulates the skin’s barrier function and homeostasis [18]. Under physiological conditions, the skin microbiota has a mutualistic relationship with the host, contributing to cutaneous homeostasis and protection against pathogens [19,20]. However, an imbalance in this relationship can lead to dysbiosis, which is implicated in the pathogenesis of numerous skin diseases, including AD [21,22]. An increase in *S. aureus* colonization in AD patients often coincides with a decrease in specific skin commensals, such as *S. epidermidis* and *C. acnes* [22]. In the context of alternative therapeutic strategies, bacteriotherapy using live bacteria from the skin microbiota has emerged as a potential treatment for inflammatory skin diseases [3,23,24]. This study aimed to assess whether structural modifications of bacterial EVs (BEVs) are capable of blocking/attenuating their pathogenic potential.

To this end, we evaluated the efficacy of a wall fragment derived from the patented strain of *C. acnes* DSM28251 (provided by Aileens Pharma S.r.l., Nova Milanese, MB, Italy). This wall fragment, referred to as c40 (DEPOSIT: 9517149, Aileens Pharma S.r.l., Nova Milanese, MB, Italy), was tested both independently and in combination with a mucopolysaccharide carrier, specifically hyaluronic acid (HAc40) (DEPOSIT: WO2022243558A1, Aileens Pharma S.r.l., Nova Milanese, MB, Italy) [25,26,27,28], to counteract the pathogenic potential of extracellular vesicles (EVs) produced by *S. aureus* ATCC 14458.

## 2. Materials and Methods

### 2.1. Chemicals and Reagents

Tryptic soy broth was purchased from Biolife and prepared according to manufacturer instructions (30 g/L, pH 7.2). Hyaluronic acid (HA) with medium molecular weight (0.50 × 106 DA) was provided by Xi’an Rongsheng Biotechnology Co., Ltd. (Xi’an, China). HA was dissolved in distilled water at 0.5 mg/mL. c40 purified bacterial fragments of *C. acnes* DSM28251 and HAc40 were used at 25 µg/mL and 0.5 mg/mL, respectively [25]. α-Hemolysin from *Staphylococcus aureus* was purchased from Sigma-Aldrich (Merck Spa) Milano Italy (cat. No. H9395), BEVs were quantified by determining the protein concentration using the Bradford assay.

### 2.2. Bacterial Growth, Bacterial Suspensions, and Free Cells Supernatant (FCS) Preparation

Cultivation of *S. aureus* ATCC14458 was performed aerobically on a rotary shaker (120 rpm) at 37 °C overnight in tryptic soy broth until stationary phase (OD600 ≥ 1). Free Cells Supernatant (FCS) was prepared as follows: The supernatant fraction was collected by centrifugation (6000× *g*, 15 min, 4 °C; and 10,000× *g*, 15 min, 4 °C). Then the supernatant was filtered through a 0.45 μm filter (to remove any remaining cells).

### 2.3. BEVs Isolation

BEVs isolation was carried out by a polymer-based methods of precipitation [29] as proposed by Wei et al. [30] with modifications. Initially, a five-fold concentrated (5×) stock solution of 1.6 M 3-[N-Morpholino] Propane Sulphonic Acid (MOPS) combined with 0.15 M sodium chloride was prepared. This solution was added to a fifteen-concentrated volume of *S. aureus* FCS, and pH was adjusted to near neutrality. Next, a stock solution of PL was prepared by dissolving PL in deionized H_2_O at a concentration of 10 mg/mL. The PL stock solution was then added to the bacterial culture medium (obtained as described above) to achieve a final concentration of 100 μg/mL. The mixture was rocked on a shaker for 45 min at room temperature to facilitate vesicle adhesion. Following incubation, the mixture underwent centrifugation at 10,000× *g* for 15 min. The resulting pellet was washed twice with PBS buffer and resuspended in a re-suspension buffer containing 50 mM Tris-HCl and 0.5 M NaCl at pH 8.5. Subsequently, the resuspended sample was subjected to ultrafiltration using 100 kDa ultrafiltration tubes (Amicon^®^ Ultra-4 cat. No. UFC9100 Merck Spa, Milano, Italy), during which PBS replaced the buffer. Finally, the isolated BEVs were stored at 4 °C until further use. The extracted BEVs were quantified by determining the total protein concentration using the Bradford assay following the manufacturer’s protocol.

### 2.4. Proteomic Analysis of BEVs by SDS Page and LC-MS/MS

Isolated BEVs were characterized by protein composition [29] For the SDS page, BEVs were suspended in the Laemmli buffer and boiled at 99 °C for 3 min before being separated on an 8% polyacrylamide gel. The gel was stained with Coomassie Brilliant Blue for 3 h and then destained overnight at room temperature. Subsequently, the bands were examined using the Gel Analyzer software 23.1.1 (available at www.gelanalyzer.com accessed on 12 February 2024 by Istvan Lazar Jr., PhD and Istvan Lazar Sr., PhD, CSc).

Filter-Aided Sample Preparation (FASP) of BEV proteins was performed by using an adapted protocol of Sielaff et al. [31] Briefly, 50 μg of proteins were transferred onto a Nanosep 10-kDa-cutoff filter (Pall Corporation, Portage, MI, USA) previously rinsed with 100 μL of 1% (*v*/*v*) formic acid (FA), the filter was washed two times with 200 μL urea buffer (8 M urea and 100 mM Tris, pH 8.5 in Milli-Q water) to remove the detergents present in the extraction buffer. 100 μL of dithiothreitol (DTT) solution (8 mM DTT in urea buffer) was added to the filter unit, covered with tin foil, and incubated for 15 min at 56 °C in a dry block heating system to reduce proteins. After two washes with 100 μL of urea buffer, the proteins were alkylated by adding 100 μL of 2-iodoacetamide (IAA) solution (50 mM IAA in urea buffer) and incubating in the dark for 20 min at room temperature. 100 μL of ammonium bicarbonate (AB) buffer (50 mM in milliQ water) was added to the filter for the buffer exchange. The digestion was performed with trypsin: a trypsin stock solution (1 μg/μL) was prepared, dissolving the enzyme in 50 mM acetic acid; 50 μL of trypsin working solution (0.01 μg/μL in AB buffer) was added onto the filter and incubated overnight at 37 °C in a wet chamber. The peptide mixture was collected by centrifugation and acidified with 10% (*v*/*v*) trifluoroacetic acid (TFA) to a final TFA concentration of 0.2% (*v*/*v*). The peptides obtained with the FASP protein digestion were analyzed on a nanoflow liquid chromatography (nLC) system (Ultimate 3000 UHPLC) coupled to an Orbitrap Fusion mass spectrometer (Thermo Fisher Scientific, Waltham, MA, USA), operating in positive ionization mode with spray voltage set at 1.7 kV and source temperature at 275 °C, equipped with a nanoEASY-Spray ion source. For the EASY-nLC system, solvent A consisted of 100% water and 0.1% formic acid; solvent B was 80% acetonitrile, and 0.1% formic acid was used. Samples were loaded on a PepMap100 C18 pre-column cartridge (5 µm particle size, 100 Å pore size, 300 µm i.d. × 5 mm length, Thermo Fisher Scientific, Waltham, MA, USA) and separated on an EASY-Spray PepMap RSLC C18 column (75 μm i.d., 3 μm particle size, 100 Å pore size, Thermo Fisher Scientific, Waltham, MA, USA) at a flow rate of 300 nL/min and a temperature of 40 °C. The gradient was as follows: from 2% to 10% B in 20 min, from 10% to 25% B in 20 min, from 25% to 40% B in 5 min, and from 40% to 90% B in 1 min, with total LC runtime of 60 min. Full mass scans (MS1)—with a mass range of *m*/*z* 350–1500, automatic gain control of 4.0 × 105, and a maximum injection time of 50 ms—were recorded in Orbitrap (resolving power of 120 K at *m*/*z* 200). Data-dependent MS/MS (MS2) analysis was performed in top speed mode with a 3s cycle time, where the most abundant multiple-charged (2+–5+) precursor ions were selected for activation in order of abundance and detected in linear ion trap at rapid scan rate. Quadrupole isolation with a 1.6 *m*/*z* isolation window was used, and dynamic exclusion was enabled for 30 s after a single scan. For MS2, automatic gain control was 1.0 × 103, and the maximum injection time was 35 ms. The fragmentation mode was the Higher-energy collisional dissociation (HCD) with 30% normalized collision energy. Data were acquired with Xcalibur software v4.4 (Thermo Fisher Scientific, Waltham, MA, USA) and processed with Peaks PEAKS studio Xpro21 (Bioinformatics Solutions Inc., Waterloo, ON, Canada) using the ‘correct precursor only’ option. Spectra were matched against the *S. aureus* STAAC database download from the Uniprot website, to which a list of common contaminants was appended (2932 entries). The false discovery rate (FDR) was set to 0.5% at the peptide-spectrum matches (PSM) level. The post-translational modification (PTM) profile was set as follows: fixed cysteine carbamidomethylation (ΔMass: 57.02), variable methionine oxidation (ΔMass: 15.99). Non-specific cleavage was allowed to one end of the peptides, with a maximum of 2 missed cleavages and Trypsin enzyme specificity. The highest error mass tolerances for precursors and fragments were set at 10 ppm.

### 2.5. BEVs Treatment with HAc40, HA, and c40

Stock solutions were prepared as previously described [25]. BEVs were treated as follows: a known concentration of BEV (20 ng) was brought into contact with HAc40, HA, and c40 (20 mg/mL) at a final concentration of 1:1 (*v*/*v*) for 2 h at room temperature under agitation at 200 rpm. The untreated BEVs (control) were placed in contact with PBS and processed as previously mentioned. All treatments were carried out within 24 h after extraction, prolonged storage at 4 °C was avoided.

### 2.6. Dynamic Light Scattering Method

The particle size and distribution analysis was conducted using the Dynamic Light Scattering (DLS) method. In short, samples containing BEV alone and treated with HA, c40, and HAc40 were diluted 1:20 in PBS pH 7.4 and allowed to equilibrate at 25 °C before analysis. Subsequently, they were analyzed using a Zetasizer Nano ZS from Malvern by Dynamic Light Scattering (DLS), also known as Photon Correlation Spectroscopy (PCS). The Z average was recorded as the intensity-weighted mean hydrodynamic size of the ensemble collection of particles measured by DLS and derived from a Cumulants analysis of the measured correlation curve, wherein a single particle size is assumed, and a single exponential fit is applied to the autocorrelation function.

### 2.7. Treated BEVs on HaCaT Cells

HaCaT keratinocytes were cultured in Dulbecco’s modified Eagle’s medium DMEM (cat. No. D5671 Sigma-Aldrich (Merck Spa), Milano, Italy.), supplemented with 10% fetal bovine serum (FBS), 1% pen/strep, and 1% L-glutamine (2 mM), in a 95% humidified atmosphere containing 5% CO_2_. The cultures were set up in T75 flasks until the cells reached about 95% confluence. The cell mat was then detached with trypsin-EDTA, resuspended in the culture medium seeded in a 6-well plate, and incubated for 48 h under the same growth conditions [32].

Finally, the wells were washed twice with phosphate-buffered saline PBS resuspended with DMEM without antibiotics and inoculated with BEV (10 ng), HAc40 HA, and c40 BEV-treated and alone.

### 2.8. Microscopic and Image Analysis for Adherent Cell Evaluation

Eosin staining of HaCaT cells was performed as follows: cells were washed with PBS and then fixed with 70% ethanol for 15–20 min. Afterward, a 0.1% eosin solution was added for 2 min. Excess eosin was removed by washing with distilled water. Finally, the stained cells were observed under a microscope.

Adherent cells were assessed using a combination of microscopic evaluation and image analysis. Images were acquired with an Olympus imt-2 inverted microscope equipped with Optech optical technology ish300 camera. The 6-well plate was positioned on the microscope stage. Subsequently, each well underwent photography using a 100× objective lens and a digital camera to acquire high-resolution images. Utilizing image analysis software, the circular area of each well was partitioned into a predefined number of equivalent sections or sectors. This segmentation was meticulously designed to ensure consistent coverage across the well area and capture representative regions suitable for subsequent analysis. Subsequently, the images of each well, delineated into uniform segments, underwent thorough analysis employing advanced image processing techniques to quantify the area contained within each sector. The area was quantified manually on ImageJ/FIJI v1.52p software using raw, unprocessed images. Finally, statistical analysis was conducted to compare the areas across different treatments.

### 2.9. MTT Assay

HaCaT cell viability alone and inoculated with BEV (10 ng per well), HAc40 c40, and HA BEV-treated and alone was evaluated by 3-[4,5-dimethylthiazol-2-yl]-2,5-diphenyl tetrasodium bromide (MTT) assay. Tests were performed as previously described with modifications [33]. Briefly, HaCaT cells were seeded in 96-well plates at a density of 1.5 × 104 per well and incubated overnight at 37 °C before experiments. Successively, cells were treated with BEV (10 ng), HAc40 c40, and HA BEV-treated and alone for 24 h. After the incubation period, 10 μL of MTT reagent (5 mg/mL) was added to each well, and the cells were incubated for 3 h at 37 °C. The formazan crystals were solubilized with 100 μL. Dimethyl sulfoxide DMSO and plates were shaken for 10 min. The absorbance was measured at 570 nm. The tests were performed in triplicate in three independent experiments.

### 2.10. RT-qPCR TJP1 ZO1 and Claudin 1

Cells were washed three times with DPBS to extract total RNA and then resuspended in Trizol reagent (Invitrogen, Waltham, MA, USA) according to the manufacturer’s protocol. cDNA was synthesized from total RNA using an iScript cDNA synthesis kit (Bio-Rad, Hercules, CA, USA). To quantify the mRNA expression level, RT-qPCR amplification was conducted using a CFX Connect real-time PCR detection system (Bio-Rad), and the PCR products were detected with SYBR Premix Ex II (TaKaRa BIO Inc., Saint-Germain-en-Laye, France). Homo sapiens ZO1, claudin 1 (CLDN1), and glyceraldehyde-3-phosphate dehydrogenase (GAPDH) primers’ sequences were retrieved from PrimerBank database (Supplementary Table S1) [34]. GAPDH was assumed to be the housekeeping gene for normalization, and the delta-delta Ct method was used for data analysis. The following thermocycling program was employed: 50 °C 2 min; 95 °C 2 min; 35 Cycles: 95 °C 15 s; annealing 60 °C 1 min; 72 °C 1 min; Melting: from 50 °C to 99 °C raising by 1 °C 5 s.

### 2.11. Statistical Analysis

Analysis of HaCaT cell BEV treatment, microscopic and image analysis, MTT assay, and RT-qPCR TJP1 ZO1 and CLDN1 were performed at least three times independently, and the data are shown as means ± DS. One-way analysis of variance (ANOVA) using Tukey’s multiple-comparison test was applied to differentiate between groups. Statistical analyses were performed using SPSS Version 26.0. Armonk, NY, USA: IBM Corp.

## 3. Results

### 3.1. Protein Analysis

**Figure 1:**
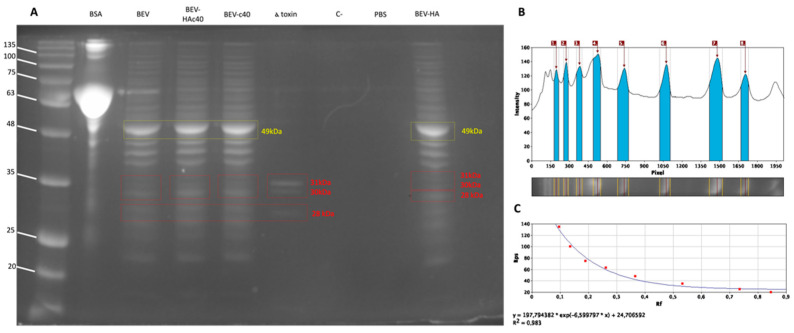
(**A**) SDS-PAGE with Coomassie blue staining (kDa), ladder: Opti-Protein XL Marker, BSA: bovine serum albumin, BEV: PL extracted *S. aureus* BEV, BEV-HAc40: BEV treated with HAc40, BEV-c40: BEV treated with c40, α toxin: *S. aureus* α-hemolysin, C−: growth media devoid of bacterial growth, PBS: phosphate buffer loaded and BEV-HA: BEV treated with HA only; (**B**) spectrophotometric analysis of BEV bands; blue columns on the graph from 1 to 8 correspond to the maximum intensity peaks detected (in pixel) for the ladder (subfigure **A**), 1: 135 kDa, 2: 100 kDa, 3: 75 kDa, 4: 63 kDA, 5: 48 kDa, 6: 35 kDa, 7: 25 kDa, 8: 20 kDa. (**C**) Standard calibration curve.

The protein profiles of isolated BEVs were evaluated by the SDS-PAGE method (Figure 1A). The Opti-Protein XL Marker from Applied Biological Materials Inc. was the standard. As a positive control, 4 μg of BSA (bovine serum albumin) was loaded, while 7 μg of untreated (BEV) and treated (BEV-HAc40, BEV-c40 and BEV-HA) samples along with *S. aureus* α-hemolysin (α-toxin) were loaded to evaluate BEVs proteins content. Finally, only growth media devoid of bacterial growth and PBS were used as negative controls (C− and PBS). Spectroscopic analysis of the isolated BEV bands was conducted to determine their molecular weight (Figure 1B), revealing the presence of a maximum peak corresponding to the band with a molecular weight of 49 kDa. A standard calibration curve was plotted To ensure the reliability of the analysis (Figure 1C).

SDS-PAGE was used to assess the protein profiles of PL-isolated EVs, (Figure 1A). Similar protein profiles, as reported by Wei et al., were retrieved in gel pattern motion of both treated and untreated samples, with an intense band at 49 kDa [30]. Moreover, the comparison of the samples with alpha-hemolysin revealed bands of the same height (31, 30 and 28 kDa), suggesting the presence of the toxin.

A proteomic study was carried out by liquid chromatography-tandem mass spectrometry to determine the protein content. A total of 422 proteins were found (Supplementary File S2 (Table S1)). According to the GO annotation (Supplementary File S2 (Table S2)), the localization of 178 proteins was identified (Supplementary File S2 (Table S3)) (42.18%), the majority had a cytoplasmatic (22.51%) and membrane localization (10.90%) while only the 5.92% were from the extracellular region (Table 1), which is consistent with findings from earlier literature [30]. A number of these were involved in the biosynthesis and degradation of cell walls (lipoteichoic acid synthase [GO:0005886], autolysin [GO:0005576] [GO:0016020]), adhesins (autolysin/adhesin Aaa, and extracellular matrix protein-binding adhesin Emp), metabolic enzymes (triacylglycerol lipase, D/L-lactate dehydrogenase, Riboflavin synthase GO:0009349], and Formate acetyltransferase [GO:0005737]), transporter proteins (SecD/SecF [GO:0005886] and ABC-type siderophore binding protein), immune evasion factors, and toxins (Alpha-hemolysin [GO:0005576], gamma-hemolysin [GO:0005576], leukocidin [GO:0005576], Delta-hemolysin extracellular [GO:0005576] [GO:0020002] [GO:0005886], Extracellular adherence protein, Fibrinogen-binding protein [GO:0005615], Staphylokinase [GO:0005615], Surface-associated serine-aspartate repeat protein D and E [GO:0005576] [GO:0016020], Staphylococcal protein A [GO:0016020], enterotoxin type O [GO:0005576]) (Supplementary File S2 (Table S2)).

Additionally, we contrasted all the 422 retrieved proteins with a public available dataset of BEV markers from *S. aureus* subsp. aureus NCTC 8325 [35], finding 77 entries (Supplementary File S2 (Table S4)). It has yet to be confirmed if these proteins co-precipitate with the vesicle or are exported inside BEVs.

### 3.2. Dynamic Light Scattering Method (DLS)

DLS provides insights into particle sizes by subjecting the particles to laser illumination and analyzing the fluctuations in scattered light intensity. The primary distribution derived from DLS is the intensity distribution, which can be converted into number and volume distribution. For our purposes, we focused on the volume distribution. Moreover, DLS was employed to determine the particle distribution’s polydispersity index (PDI). PDI serves as an indicator of the breadth of the distribution, with values less than 0.3 indicating a narrow size distribution and values exceeding 0.5 indicating a broader distribution of particles [36,37].

**Figure 2:**
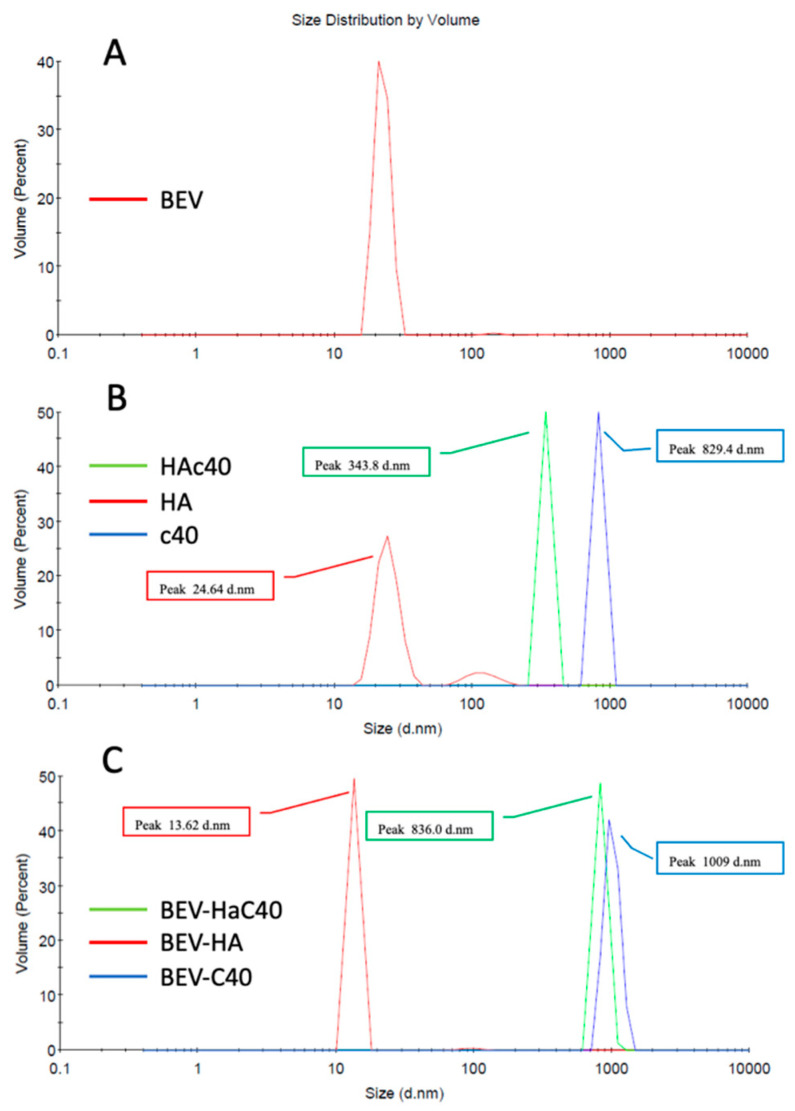
(**A**) Analysis using Zetasizer Nano ZS of BEVs isolated from *S. aureus*; (**B**) Analysis using Zetasizer Nano ZS of HA, HAc40, c40; (**C**) Analysis using Zetasizer Nano ZS of BEV-HA, BEV-HAc40, BEV-c40.

*S. aureus* BEVs size (Figure 2A), as well as the particle size of HAc40, HA, and c40, in PBS pH 7.4, have been evaluated (Figure 2B). Additionally, the effects on the size distribution of these substances on BEVs have been reported in Figure 2C. The analysis focused on the average size of the particle populations and the percentage of the most prevalent particles in volumetric terms by assessing the peaks of the particle volumes. Treated BEVs change, increasing or decreasing their size. In particular, BEV-HA decreased their size, shifting from a peak of 22.45 nm to 13.62 nm. These variations are consistent with the analysis of the average sizes of the respective populations observed by intensity, which decreased from 312.3 nm to 58.14 nm, respectively. Conversely, BEV-HAc40 and BEV-c40 showed an increase in peak size, rising from 22.45 nm to 836.0 nm for HAc40 and to 1009 nm for c40, with average sizes of the observed populations varying from 312.3 nm to 1090 nm and 2379 nm, respectively (Figure 2B,C). Lastly, HA, HAc40, and c40 alone dissolved in PBS showed respectively the following volumetric peaks: HA 24.64 nm; HAc40 343.8 nm; c40 829.4 nm, while the analysis of the average particle size exhibited the following values: HA 81 nm; HAc40 825 nm; c40 1799 nm (Table 2 and Supplementary File S3).

### 3.3. Assessment of Adherent Cells by Microscopic Evaluation and Image Analysis

**Figure 3:**
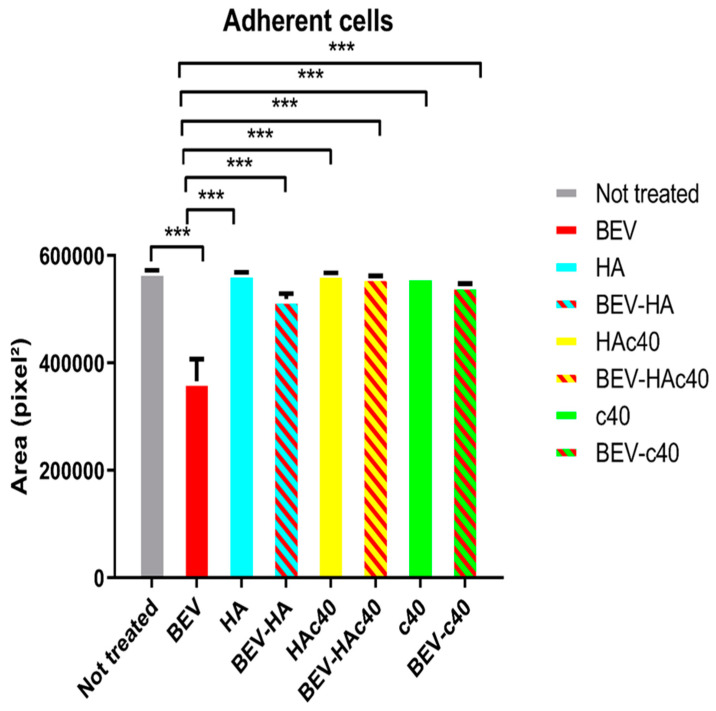
Assessment of adherent cells by microscopic evaluation and image analysis. Mean area (pixel^2^) with standard deviation (*** *p*-value < 0.001).

**Figure 4:**
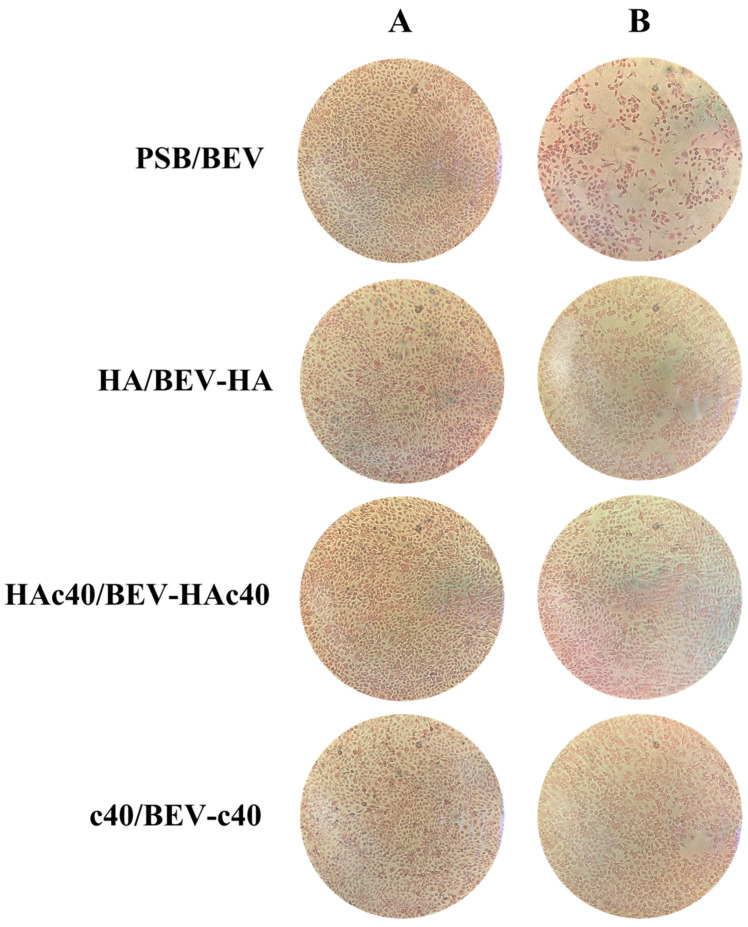
Microscopic images of in vitro treated HaCaT cells stained with eosin (40× magnification). Column A cells not exposed to BEV: PBS Control, HA treatment, HAc40 and c40 treatment. Column B cells exposed to BEV: BEV, BEV exposed to HA (BEV-HA), BEV exposed to HAc40 (BEV-HAc40), BEV exposed to c40 (BEV-c40).

Figure 3 and Table 3 present the mean area (pixel^2^) with standard deviation, and the mean percentage difference in area between treated and untreated groups expressed as a percentage of the treatment mean. Additionally, the *p*-value for statistical significance is provided. For the BEV treatment, the mean area is 366,445 pixel^2^ with a standard deviation of ±15.26. The mean percentage difference in area compared to the untreated (UT) group is −55.83%, with a *p*-value < 0.001, indicating a significant reduction in area compared to the untreated group with PBS. The mean area for the BEV HA treatment is 519,178.66 pixel^2^ with a standard deviation of ±14.31. The mean percentage difference in area compared to the BEV treatment is 29.42%, indicating an increase in area compared to the BEV treatment. The mean area for the BEV c40 treatment is 545,091.33 pixel^2^ with a standard deviation of ±13.76. The mean percentage difference in area compared to the BEV treatment is 32.77%, indicating an increase in area compared to the BEV treatment. The mean area for the BEV HAc40 treatment is 561,058.66 pixel^2^ with a standard deviation of ±17. The mean percentage difference in area compared to the BEV treatment is 34.69%, indicating an increase in area compared to the BEV treatment. These findings suggest that the BEV HAc40 treatment yields the most significant increase in area compared to other treatments. In Figure 4, a representative image for each condition of the eosin-stained cells is shown.

### 3.4. MTT Assay

**Figure 5:**
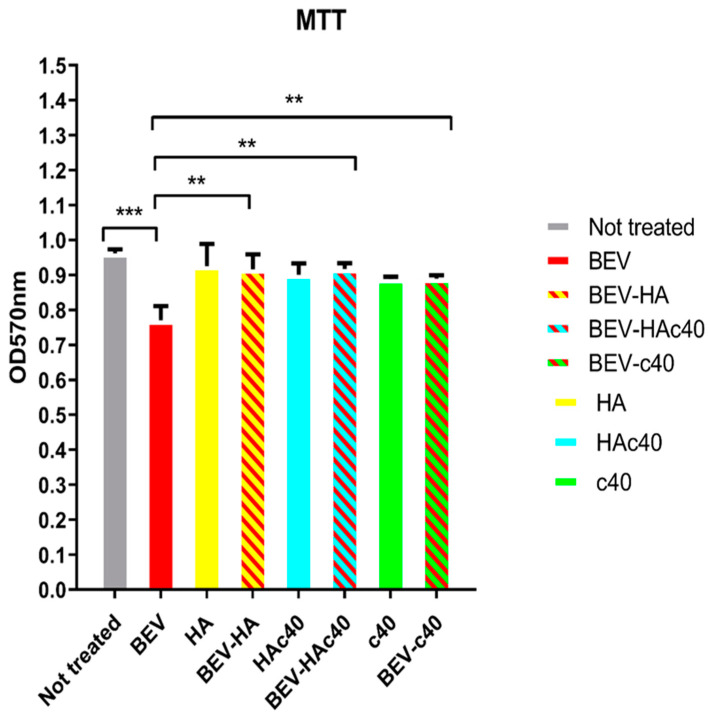
MTT assay results show significant differences in cell viability among treatment groups. BEV-treated cells exhibit reduced viability compared to untreated cells (q = 9.873, *p* ≤ 0.001, ***), as well as HA-treated cells (q = 7.988, *p* ≤ 0.001 ***). Significant differences are observed in comparisons with BEV-HA, BEV-Hac40 and BEV-c40 treated cells (*p* ≤ 0.01 ** or ≤0.001 ***). No significant differences in cell viability are noted within pairs of treatments such as HA vs. BEV-HA, HAc40 vs. BEV-Hac40, and c40 vs. BEV-c40 (*p* > 0.05, ns).

The MTT assay (Figure 5) reveals significant differences in cell viability among the treatment groups. The comparison between untreated cells and those treated with BEV shows a mean difference of 0.192, indicating a substantial decrease in cell viability in the BEV-treated group (q = 9.873, *p* ≤ 0.001, ***). This finding underscores the cytotoxic effects of BEV on HaCaT cells. Comparing BEV-treated cells with those treated with HA reveals a mean difference of −0.1553, showing a significant reduction in cell viability in the BEV group compared to the HA group (q = 7.988, *p* ≤ 0.001, ***). Similarly, when BEV-treated cells are compared to those treated with BEV-HA, BEV-Hac40, and BEV-c40, all comparisons yield significant differences in cell viability, highlighting the protective effects of these treatments against BEV-induced cytotoxicity (*p* ≤ 0.01 ** or ≤0.001 ***). Notably, there are no significant differences in cell viability between HA vs. BEV-HA, HAc40 vs. BEV-Hac40, and c40 vs. BEV-c40 (*p* > 0.05, ns), indicating comparable effects on cell viability within these pairs of treatments.

### 3.5. mRNA Expression Levels of ZO-1 and CLDN1

**Figure 6:**
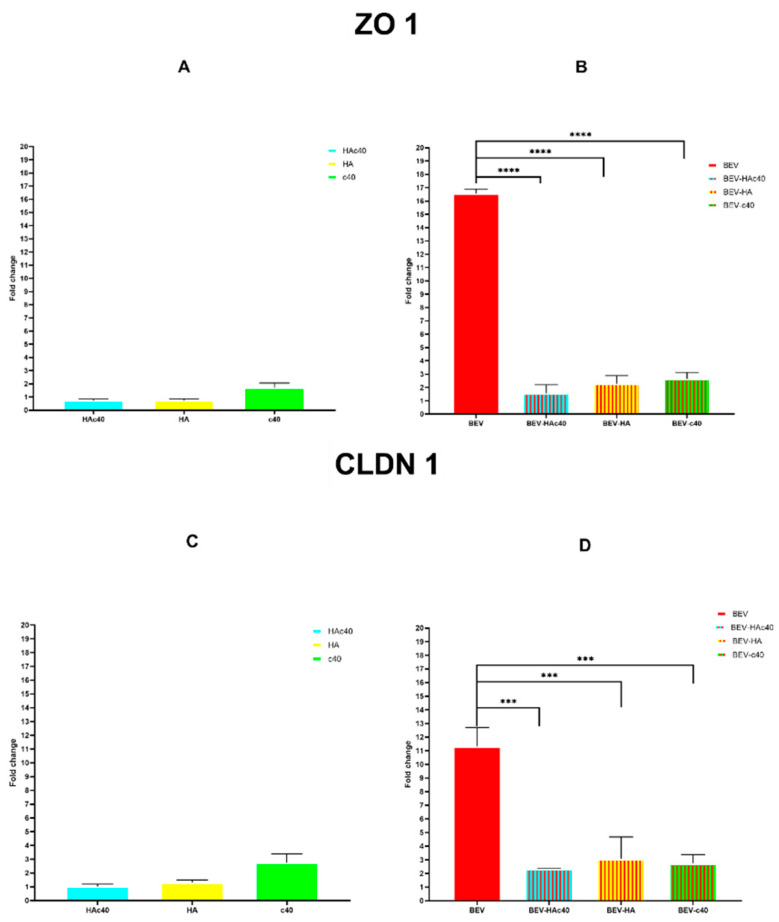
RT-qPCR Tight Junction expression, normalized by GAPDH as housekeeping gene and analyzed with delta delta Ct method. (**A**) ZO1 gene fold expression for HAc40 HA and c40 treated HaCaT cell line. (**B**) ZO1 gene fold expression for BEV BEV-HAc40 BEV-HA and BEV-c40 treated HaCaT cell line. (**C**) CLDN1 gene fold expression for HAc40 HA and c40 treated HaCaT cell line. (**D**) CLDN1 gene fold expression for BEV BEV-HAc40 BEV-HA and BEV-c40 treated HaCaT cell line. Statistical analysis significance results (*p*-value) obtained by oneway ANOVA comparing gene expression levels between BEV alone and BEV-HAc40 BEV-HA and BEV-c40 treated for both CLDN1 and ZO1 was included as follows: *** *p* ≤ 0.001, **** *p* ≤ 0.0001.

The mRNA levels of CLDN1 and ZO-1 in HaCaT cells treated with HAc40, HA, and C40 alone did not show significant changes (Figure 6A–C). However, in contrast, BEV treatment BEV led to a marked increase in the expression of both ZO1 and CLDN1, with fold changes of 16.57 ± 0.325 and 11.30 ± 1.41, respectively (Figure 6B–D).

Further analysis revealed that the mRNA levels of ZO-1 in the BEV-HAc40 were significantly reduced to 1.56 ± 0.11 with a fold change reduction of 15.84 (SE of difference 0.4248) compared to BEV alone, mirroring levels similar to those seen with HAc40 treatment alone (0.73 ± 0.11). Similar results were observed for BEV-HA and BEV-c40, with reductions of 14.30 and 13.93 (SE of difference 0.4248), respectively, compared to BEV treatment alone (Figure 6A,B).

Lastly, mRNA levels of CLDN1 showed a comparable trend to that of ZO1. Indeed, expression fold in the BEV-HAc40 group was reduced to 2.34 ± 0.02 with a fold change reduction of 8975 (SE of difference 0.8815), while in the BEV-HA and BEV-c40 groups, the reductions were 8.26 and 8.58 (SE of difference 0.8815), respectively, compared to BEV treatment alone (Figure 6C,D).

## 4. Discussion

*S. aureus* is known to release vesicles into the extracellular environment. These EVs are spherical, closed structures with a double lipid layer with a diameter ranging from 20 to 100 nm. EVs play a significant role in the pathogenesis of infections in the human host, including AD [3].

EVs isolated from *S. aureus* ATCC 14,458 have been found to contain toxins that may be associated with increased severity of skin lesions in AD patients, as demonstrated in the study conducted by Jun et al. [11] These vesicles contain components, including membrane lipids such as phospholipids, fatty acids, and sterols, nucleic acids, such as 16S rRNA-encoding DNA, sugars, enzymes, and cytoplasmic proteins. Proteomic analysis conducted by Lee et al. revealed 90 vesicular proteins, particularly enriched with extracellular virulent or surface-associated ones [10,11]. Findings reported in this paper about EVs isolated from *S. aureus* ATCC 14,458 were found to agree with data from the literature as well as data submitted in public databases about *Staphylococcus aureus subsp. aureus* NCTC 8325 (Supplementary File S2 (Table S4)) [35]. The protein profile retrieved from SDS-PAGE, as shown in Figure 1, is consistent with the results published by Wei et al. [30]. Furthermore, the treatment of BEV with HAc40, c40, or HA does not appear to alter their protein profile, including the bands corresponding to alpha toxin present in all samples.

Additionally, the 178 proteins in this study, categorized by GO annotation using the biological functional hierarchy (Table 1), have locations and functions consistent with those of previously published research [10]. The investigation also found several immune responses key-evasion factors, and toxins present [11]. Additionally, two transporter proteins, SecD/SecF translocase protein, and ABC-type siderophore binding protein, were found to be involved in the translocation of proteins or iron ions that may help transfer proteins to their own or other bacteria as described for Gram-negative bacteria EVs [38] (Supplementary File S2 (Tables S1–S3)). Overall, proteomic analyses conducted on *S. aureus* BEVs provided marker characterization as required by Welsh et al. [29]

To our knowledge, this is the first in vitro study that attempts to define the relationship between EV size and pathogenicity. Despite the lack of pertinent literature, studies on phospholipid-based particle offer a plausible explanation of our findings due of their structural similarities to EVs. These particles exhibit distinct behaviors based on size: large vesicles, equal to or greater than 600 nm, have difficulty to penetrate deeper skin layers. Conversely, liposomes sized at or below 300 nm show some ability to deliver their contents into deeper skin layers. Noteworthy, liposomes with a size of 70 nm (or less) show potential for dermal delivery both in the viable epidermis and the dermis. Nanoparticles smaller than 6–7 nm in size can pass the lipidic transepidermal routes, while those smaller than 36 nm can pass through the aqueous pores. Particles falling within the size range of 10–210 nm may preferentially penetrate through the trans follicular route [36,39].

Furthermore, particle size is a critical factor in determining cellular absorption and subsequent interaction with cellular components. The intracellular trafficking pathways of particles, their likelihood of being identified by particular cellular receptors, and their ability to cross cellular membranes are all directly influenced by size. Larger particles may struggle to pass through cellular barriers effectively, leading to altered internalization kinetics or reduced cellular uptake. Conversely, smaller particles can more easily pass through cell membranes, often resulting in increased cellular absorption [40].

These findings demonstrate that treatment with c40 or HAc40 significantly increased *S. aureus* BEV size from a peak particle size of 22.45 nm to 836 and 1009 nm, respectively (Table 1 and Figure 2). This considerable enlargement may make it more difficult for these vesicles to interact with biological components, potentially impacting the release of their contents. These changes appear to be a result of altering the particles rather than simply combining two distinct particle populations. The volumetric changes due to EVs treatment with c40 and HAc40 are more significant than those observed in the individual populations of BEVs, c40, and HAc40. These data suggest an interaction between EV and the active substances; indeed, if there was only mixing during treatment, a downward change in the diameter of the largest particles (i.e., c40 and HAc40) should be expected. Furthermore, the average particle population of HA-treated BEVs is lower than the smallest value between HA or BEV alone, suggesting that HA directly acted on the BEVs, and the new result is not likely due to the mixture of two different particle populations (Figure 2B,C).

The findings from the NTA analysis are supported by in vitro investigations into the pathogenicity of BEVs. The immortalized keratinocyte cell line HaCaT was selected for in vitro experiments due to its similarity to primary keratinocyte cultures and previous use in studies of *S. aureus*-keratinocyte interactions [41,42].

Microscopic evaluation and image analysis of adherent cells (Figure 3 and Figure 4 and Table 1) suggest that the BEV HAc40 treatment resulted in the most significant increase in area compared to other treatments. Moreover, MTT assay results indicated the significant protective effects of HA, HA/BEV, HAc40, HAc40/BEV, c40, and c40/BEV treatments against BEV-induced cytotoxicity, as shown by differences in cell viability compared to the BEV-treated group (Figure 5).

Regarding mRNA expression of the tight junctions ZO1 and CLDN1, in vitro treatment with HAc40, c40, or HA of BEV-uninfected cells did not affect the fold change of either gene tested (Figure 6A–C). However, treatment with BEVs alone resulted in a significant increase in the expression of both ZO1 and CLDN1 genes, with fold changes of 16.57 ± 0.325 and 11.30 ± 1.41, respectively (Figure 6B–D). These findings, although the first reported on the in vitro effects of S. aureus EVs, can be explained by existing literature. Kwak et al. observed an increase in ZO-1 mRNA levels in the CACO-2 cell line following exposure to *S. aureus* alpha-toxin, without a corresponding increase in protein expression. This suggests either missed translation or mRNA degradation [43].

Furthermore, Ohnemus et al. confirmed these findings with an in vitro infection model. Investigation on the HaCaT cell line with different *S. aureus* strains negative for exfoliative toxin also showed an early increase in ZO-1 mRNA levels upon infection [44]. Treatment with HA, HAc40, or c40 of BEV-infected cells drastically reduces the overexpression of both genes, with a greater reduction observed for HAc40 with a fold change reduction of 15.84 (SE of difference 0.4248) and 975 (SE of difference 0.8815) for ZO1 and CLDN1 respectively (Figure 6B–D). These data agree with those previously reported by Magnifico et al. on an ex vivo model of pig skin infected with *S. aureus* and treated with HAc40 and C40. The *stratum corneum* was damaged in tissues infected with two strains of *S. aureus*, as demonstrated by the immunohistochemically detected reduction in Claudin-1 and ZO-1 expression signals. On the other hand, administering c40 and HAc40 treatments led to elevated signal levels comparable to those observed in the control group [25].

## 5. Conclusions

To sum up, AD is a complex skin condition characterized by immunological dysregulation, compromised barrier function, and microbial colonization, particularly by *S. aureus*. This study has clarified the complex interactions between BEV, host cells, and a potential therapeutic strategy in the context of AD pathogenesis. The study also highlights the potential efficacy of *C. acnes*-derived fragments, specifically HAc40 and C40, in reducing the cytotoxicity and barrier dysfunction caused by *S. aureus* EVs. Dynamic light scattering and in vitro cell viability experiments showed that treated *S. aureus* EVs changed significantly in size and pathogenicity. Although the size variability and/or modification of *S. aureus* extracellular vesicles and their potential pathogenicity in vitro on HaCaT keratinocyte cell line are documented for the first time in this manuscript, the study has some limitations.

Among these is our lack of comprehensive understanding regarding the possible processes by which HAc40 or c40 influence both *S. aureus* BEV size and in vitro cell interaction. Transmission electron microscopy (TEM) by itself can provide information on how the treatment affects size, but it is not able to conclusively identify if these substances internalize or integrate into BEV membranes. Therefore, further fluorophores labeling research that combine confocal analysis and TEM is needed for interactions and internalization studies. This could provide detailed information on how HAc40 and c40 treatment affects BEV size and their interaction with keratinocytes in vitro, allowing the fully address of these constraints and develop an understanding of the pathogenic potential of *S. aureus* BEVs.

## Figures and Tables

**Table 1:** Gene Ontology analysis of total protein BEV content identified by LC-MS/MS analysis.

Cellular Component/s	Gene Ontology (GO)	Numbers of Proteins	% (n. 422)
TOTAL		178	42.18
Cytoplasm		95	22.51
ribonucleoprotein	[GO:1990904]	19	
small ribosomal subunit	[GO:0015935]	4	
large ribosomal subunit	GO:0015934]	7	
cell division site	[GO:0032153]	1	
DNA polymerase III complex	[GO:0009360]	1	
DNA-directed RNA polymerase complex	[GO:0000428]	2	
nucleoid	[GO:0009295]	1	
Cytoplasmatic only	[GO:0005737]	60	
Membrane		46	10.90
plasma membrane	[GO:0005886]	13	
membrane raft	[GO:0045121]	1	
proton-transporting ATP synthase complex	[GO:0045261]	3	
respirasome	[GO:0070469]	2	
membrane only	[GO:0016020]	27	
Extracellular region		25	5.92
membrane	[GO:0016020]	5	
extracellular space	[GO:0005615]	1	
host cell plasma membrane; membrane	[GO:0020002] [GO:0005886]	1	
phosphopyruvate hydratase; cell surface	[GO:0000015] [GO:0009986]	2	
Extracellular region only	[GO:0005576]	16	
DNA-directed RNA polymerase complex	[GO:0000428]	2	0.47
Glycerol-3-phosphate dehydrogenase	[GO:0009331]	2	0.47
Cellular anatomical entity	[GO:0110165]	2	0.47
Riboflavin synthase complex	[GO:0009349]	1	0.24
HslUV protease complex	[GO:0009376]	1	0.24
ABC transporter complex	[GO:0043190]	1	0.24
Chromosome	[GO:0005694]	1	0.24

**Table 2:** Peaks and Z-Average of BEV, BEV exposed to Ha, HAc40, c40 (BEV-HA, BEV-HAc40, BEV-c40) and of single substances (Ha, HAc40, c40) obtained by Zetasizer Nano ZS.

	BEV	HA	HAc40	c40	BEV-HA	BEV-HAc40	BEV-c40
Volume/Peak (nm)	22.45	24.64	343.8	829.4	13.62	836.0	1009
Z-Average (nm)	312.3	81	825	1799	58.14	1090	2379

**Table 3:** Assessment of adherent cells by microscopic evaluation and image analysis. Mean percentage difference in area (pixel^2^) between treated and untreated groups, expressed as a percentage of the treatment mean. Statistically significant values are shown in bold type.

Treated (T)	Mean Area (Pixel^2^ ± D.S.)	Untreated (UT)	Mean Area (Pixel^2^ ± D.S.)	Mean Area Percentage Difference (T-UT)/T × 100	*p*-Value
BEV	366,445 ± 15.26	PBS	571,017.33 ± 24.35	−55.83	**<0.001**
BEV-HAc40	561,058.66 ± 17	BEV	366,445 ± 15.26	34.69	
BEV-HA	519,178.66 ± 14.31			29.42	
BEV-c40	545,091.33 ± 13.76			32.77	

## Data Availability

The authors confirm that the data supporting the findings of this study are available within the article and its Supplementary Materials.

## Supplementary Materials

The following supporting information can be downloaded at: https://www.mdpi.com/article/10.3390/pharmaceutics16060789/s1, Supplementary S1 Table S1 Primer used in this study; Supplementary S2 Table S1 All protein, Table S2 GO all proteins, Table S3 GO localization, Table S4 BEV markers from *Staphylococcus aureus* subsp. aureus NCTC 8325 found in PL-BEVs; Supplementary S3 Figure S1 Analysis using Zetasizer Nano ZS of Ha, Figure S1.1 Analysis using Zetasizer Nano ZS of Bev-Ha, Figure S1.2 Analysis using Zetasizer Nano ZS of Bev, Bev-Ha, Ha, Figure S2 Analysis using Zetasizer Nano ZS of HaC40, Figure S2.1 Analysis using Zetasizer Nano ZS of Bev-HaC40, Figure S2.2 Analysis using Zetasizer Nano ZS of Bev, Bev-HaC40, HaC40, Figure S3 Analysis using Zetasizer Nano ZS of C40, Figure S3.1 Analysis using Zetasizer Nano ZS of Bev-C40, Figure S3.2 Analysis using Zetasizer Nano ZS of Bev, Bev-C40, C40.

## References

[1] Weidinger S., Beck L.A., Bieber T., Kabashima K., Irvine A.D. (2018). Atopic Dermatitis. Nat. Rev. Dis. Primers.

[2] Geoghegan J.A., Irvine A.D., Foster T.J. (2018). *Staphylococcus aureus* and Atopic Dermatitis: A Complex and Evolving Relationship. Trends Microbiol..

[3] Magnifico I., Petronio Petronio G., Venditti N., Cutuli M.A., Pietrangelo L., Vergalito F., Mangano K., Zella D., Di Marco R. (2020). Atopic Dermatitis as a Multifactorial Skin Disorder. Can the Analysis of Pathophysiological Targets Represent the Winning Therapeutic Strategy?. Pharmaceuticals.

[4] Foster T.J., Geoghegan J.A., Ganesh V.K., Höök M. (2014). Adhesion, Invasion and Evasion: The Many Functions of the Surface Proteins of *Staphylococcus aureus*. Nat. Rev. Microbiol..

[5] Iwatsuki K., Yamasaki O., Morizane S., Oono T. (2006). Staphylococcal Cutaneous Infections: Invasion, Evasion and Aggression. J. Dermatol. Sci..

[6] Bukowski M., Wladyka B., Dubin G. (2010). Exfoliative Toxins of *Staphylococcus aureus*. Toxins.

[7] Nakatsuji T., Chen T.H., Narala S., Chun K.A., Two A.M., Yun T., Shafiq F., Kotol P.F., Bouslimani A., Melnik A.V. (2017). Antimicrobials from Human Skin Commensal Bacteria Protect against *Staphylococcus aureus* and Are Deficient in Atopic Dermatitis. Sci. Transl. Med..

[8] Hong S.-W., Choi E.-B., Min T.-K., Kim J.-H., Kim M.-H., Jeon S.G., Lee B.-J., Gho Y.S., Jee Y.-K., Pyun B.-Y. (2014). An Important Role of α-Hemolysin in Extracellular Vesicles on the Development of Atopic Dermatitis Induced by *Staphylococcus aureus*. PLoS ONE.

[9] Tam K., Torres V.J. (2019). *Staphylococcus aureus* Secreted Toxins and Extracellular Enzymes. Microbiol. Spectr..

[10] Lee E.-Y., Choi D.-Y., Kim D.-K., Kim J.-W., Park J.O., Kim S., Kim S.-H., Desiderio D.M., Kim Y.-K., Kim K.-P. (2009). Gram-Positive Bacteria Produce Membrane Vesicles: Proteomics-Based Characterization of *Staphylococcus aureus*-Derived Membrane Vesicles. Proteomics.

[11] Jun S.H., Lee J.H., Kim S.I., Choi C.W., Park T.I., Jung H.R., Cho J.W., Kim S.H., Lee J.C. (2017). *Staphylococcus aureus*-Derived Membrane Vesicles Exacerbate Skin Inflammation in Atopic Dermatitis. Clin. Exp. Allergy.

[12] Jin S.-P., Han S.B., Kim Y.K., Park E.E., Doh E.J., Kim K.H., Lee D.H., Chung J.H. (2016). Changes in Tight Junction Protein Expression in Intrinsic Aging and Photoaging in Human Skin in Vivo. J. Dermatol. Sci..

[13] Svoboda M., Bílková Z., Muthný T. (2016). Could Tight Junctions Regulate the Barrier Function of the Aged Skin?. J. Dermatol. Sci..

[14] Brandner J.M., Schulzke J.D. (2015). Hereditary Barrier-Related Diseases Involving the Tight Junction: Lessons from Skin and Intestine. Cell Tissue Res..

[15] Bäsler K., Bergmann S., Heisig M., Naegel A., Zorn-Kruppa M., Brandner J.M. (2016). The Role of Tight Junctions in Skin Barrier Function and Dermal Absorption. J. Control. Release.

[16] Yokouchi M., Kubo A. (2018). Maintenance of Tight Junction Barrier Integrity in Cell Turnover and Skin Diseases. Exp. Dermatol..

[17] Herbig M.E., Houdek P., Gorissen S., Zorn-Kruppa M., Wladykowski E., Volksdorf T., Grzybowski S., Kolios G., Willers C., Mallwitz H. (2015). A Custom Tailored Model to Investigate Skin Penetration in Porcine Skin and Its Comparison with Human Skin. Eur. J. Pharm. Biopharm..

[18] Harris-Tryon T.A., Grice E.A. (2022). Microbiota and Maintenance of Skin Barrier Function. Science.

[19] Luna P.C. (2020). Skin Microbiome as Years Go By. Am. J. Clin. Dermatol..

[20] Chen P., He G., Qian J., Zhan Y., Xiao R. (2021). Potential Role of the Skin Microbiota in Inflammatory Skin Diseases. J. Cosmet. Dermatol..

[21] Flowers L., Grice E.A. (2020). The Skin Microbiota: Balancing Risk and Reward. Cell Host Microbe.

[22] Paller A.S., Kong H.H., Seed P., Naik S., Scharschmidt T.C., Gallo R.L., Luger T., Irvine A.D. (2019). The Microbiome in Patients with Atopic Dermatitis. J. Allergy Clin. Immunol..

[23] Ito Y., Amagai M. (2022). Controlling Skin Microbiome as a New Bacteriotherapy for Inflammatory Skin Diseases. Inflamm. Regen..

[24] Zhou H., Shi L., Ren Y., Tan X., Liu W., Liu Z. (2020). Applications of Human Skin Microbiota in the Cutaneous Disorders for Ecology-Based Therapy. Front. Cell Infect. Microbiol..

[25] Magnifico I., Perna A., Cutuli M.A., Medoro A., Pietrangelo L., Guarnieri A., Foderà E., Passarella D., Venditti N., Vergalito F. (2023). A Wall Fragment of *Cutibacterium Acnes* Preserves Junctional Integrity Altered by *Staphylococcus aureus* in an Ex Vivo Porcine Skin Model. Pharmaceutics.

[26] Sacchetti R., Gregori G., Moggio E., Gobbo L., Bonzano L., Pellacani G. (2021). HAc40 Is a Novel Microbiome Modulator, Effective on Atopic Dermatitis in Children: Data from Two Pilot Vehicle-Controlled Trials. J. Eur. Acad. Dermatol. Venereol..

[27] Pietrangelo L., Dattola A., Magnifico I., Petronio Petronio G., Cutuli M.A., Venditti N., Guarnieri A., Wollenberg A., Pellacani G., Di Marco R. (2023). Efficacy and Microbiota Modulation Induced by LimpiAL 2.5%, a New Medical Device for the Inverse Psoriasis Treatment. Int. J. Mol. Sci..

[28] Pietrangelo L., Magnifico I., Guerrera A., Cutuli M.A., Petronio G.P., Venditti N., Covelli M., Buccieri N., Garofalo S., Di Marco R. (2021). LimpiAD Foam and the Potential Control of the Pressure Ulcers Onset. Biomed. Pharmacother..

[29] Welsh J.A., Goberdhan D.C.I., O’Driscoll L., Buzas E.I., Blenkiron C., Bussolati B., Cai H., Di Vizio D., Driedonks T.A.P., Erdbrügger U. (2024). Minimal Information for Studies of Extracellular Vesicles (MISEV2023): From Basic to Advanced Approaches. J. Extracell. Vesicles.

[30] Wei S., Jiao D., Xing W. (2022). A Rapid Method for Isolation of Bacterial Extracellular Vesicles from Culture Media Using Epsilon-Poly-L-Lysine That Enables Immunological Function Research. Front. Immunol..

[31] Sielaff M., Kuharev J., Bohn T., Hahlbrock J., Bopp T., Tenzer S., Distler U. (2017). Evaluation of FASP, SP3, and iST Protocols for Proteomic Sample Preparation in the Low Microgram Range. J. Proteome Res..

[32] Ngo Q.V., Faass L., Sähr A., Hildebrand D., Eigenbrod T., Heeg K., Nurjadi D. (2022). Inflammatory Response Against *Staphylococcus aureus* via Intracellular Sensing of Nucleic Acids in Keratinocytes. Front. Immunol..

[33] Nicolosi D., Genovese C., Cutuli M.A., D’angeli F., Pietrangelo L., Davinelli S., Petronio G.P., Di Marco R. (2020). Preliminary in Vitro Studies on Corynebacterium Urealyticum Pathogenetic Mechanisms, a Possible Candidate for Chronic Idiopathic Prostatitis?. Microorganisms.

[34] Spandidos A., Wang X., Wang H., Seed B. (2010). PrimerBank: A Resource of Human and Mouse PCR Primer Pairs for Gene Expression Detection and Quantification. Nucleic Acids Res..

[35] Kim D.-K., Kang B., Kim O.Y., Choi D.-S., Lee J., Kim S.R., Go G., Yoon Y.J., Kim J.H., Jang S.C. (2013). EVpedia: An Integrated Database of High-Throughput Data for Systemic Analyses of Extracellular Vesicles. J. Extracell. Vesicles.

[36] Danaei M., Dehghankhold M., Ataei S., Hasanzadeh Davarani F., Javanmard R., Dokhani A., Khorasani S., Mozafari M. (2018). Impact of Particle Size and Polydispersity Index on the Clinical Applications of Lipidic Nanocarrier Systems. Pharmaceutics.

[37] Wu L., Zhang J., Watanabe W. (2011). Physical and Chemical Stability of Drug Nanoparticles. Adv. Drug Deliv. Rev..

[38] Lee E.-Y., Choi D.-S., Kim K.-P., Gho Y.S. (2008). Proteomics in Gram-Negative Bacterial Outer Membrane Vesicles. Mass Spectrom. Rev..

[39] Verma D.D., Verma S., Blume G., Fahr A. (2003). Particle Size of Liposomes Influences Dermal Delivery of Substances into Skin. Int. J. Pharm..

[40] Augustine R., Hasan A., Primavera R., Wilson R.J., Thakor A.S., Kevadiya B.D. (2020). Cellular Uptake and Retention of Nanoparticles: Insights on Particle Properties and Interaction with Cellular Components. Mater. Today Commun..

[41] Miyake R., Iwamoto K., Sakai N., Matsunae K., Aziz F., Sugai M., Takahagi S., Tanaka A., Hide M. (2022). Uptake of *Staphylococcus aureus* by Keratinocytes Is Reduced by Interferon-Fibronectin Pathway and Filaggrin Expression. J. Dermatol..

[42] Al-Rayyan N., Steidl O., Starr N.L., Smith J., Singh A.M. (2023). Effect of *Staphylococcus aureus* on the Keratinocytes in Atopic Dermatitis. J. Allergy Clin. Immunol..

[43] Kwak Y.-K., Vikström E., Magnusson K.-E., Vécsey-Semjén B., Colque-Navarro P., Möllby R. (2012). The *Staphylococcus aureus* Alpha-Toxin Perturbs the Barrier Function in Caco-2 Epithelial Cell Monolayers by Altering Junctional Integrity. Infect. Immun..

[44] Ohnemus U., Kohrmeyer K., Houdek P., Rohde H., Wladykowski E., Vidal S., Horstkotte M.A., Aepfelbacher M., Kirschner N., Behne M.J. (2008). Regulation of Epidermal Tight-Junctions (TJ) during Infection with Exfoliative Toxin-Negative Staphylococcus Strains. J. Investig. Dermatol..

