# Supplementary material for: Targeting S. aureus Extracellular Vesicles: A New Putative Strategy to Counteract Their Pathogenic Potential
Source: Pharmaceutics. 2024 Jun 11;16(6):789. doi: 10.3390/pharmaceutics16060789 (PMC11207539; doi:10.3390/pharmaceutics16060789)
Supplement: Supplementary file 1 [file pharmaceutics-16-00789-s001.zip › Supplementary 3.docx]

**Supplementary 3**

**Ha**

Peak 26.64 nm

Z-Average 81.02 nm

Figure 1 Analysis using Zetasizer Nano ZS of Ha

**Bev-Ha**

Peak 13.62 nm

Z-Average 58.14 nm

Figure 1.1 Analysis using Zetasizer Nano ZS of Bev-Ha;

**Bev-BevHa-Ha**

Peak 22.45 nm

Z-Average 312.3 nm

Peak 24.64 nm

Z-Average 81.02 nm

Peak 13.62 nm

Z-Average 58.14 nm

Figure 1.2 Analysis using Zetasizer Nano ZS of Bev, Bev-Ha, Ha

**HaC40**

Peak 343.8 nm

Z-Average 825.7 nm

Figure 2 Analysis using Zetasizer Nano ZS of HaC40

**Bev-HaC40**

Peak 836.0 nm

Z-Average 1090 nm

Figure 2.1 Analysis using Zetasizer Nano ZS of Bev-HaC40

**Bev-BevHaC40-HaC40**

Peak 343.8 nm

Z-Average 825.7 nm

Peak 836.0 nm

Z-Average 1090 nm

Peak 22.45 nm

Z-Average 312.3nm

Figure 2.2 Analysis using Zetasizer Nano ZS of Bev, Bev-HaC40, HaC40

**C40**

Peak 829.4 nm

Z-Average 1799 nm

Figure 3 Analysis using Zetasizer Nano ZS of C40

**Bev-C40**

Peak 1009 nm

Z-Average 2379 nm

Figure 3.1 Analysis using Zetasizer Nano ZS of Bev-C40

**Bev-BevC40-C40**

Peak 1009 nm

Z-Average 2379 nm

Peak 829.4 nm

Z-Average 1799 nm

Peak 22.45 nm

Z-Average 312.3 nm

Figure 3.2 Analysis using Zetasizer Nano ZS of Bev, Bev-C40, C40

Figures 1.2, 2.2, and 3.2 showed that the exposure of BEVs to Ha, HaC40 and C40 resulted in important variations in both the size of the average particles populations (Z-Average) and in their volumetric peaks. These variations seems to stem from a modification of the BEVs rather than a mere mixing of two distinct particle populations.
