# Supplementary material for: Targeting S. aureus Extracellular Vesicles: A New Putative Strategy to Counteract Their Pathogenic Potential
Source: Pharmaceutics. 2024 Jun 11;16(6):789. doi: 10.3390/pharmaceutics16060789 (PMC11207539; doi:10.3390/pharmaceutics16060789)
Supplement: Supplementary file 1 [file pharmaceutics-16-00789-s001.zip › Supplementary 1.docx]

Table 1 Primer used in this study

| **Homo sapiens tight junction protein 1, zona occludens 1 (ZO1)** | | | | | |
| --- | --- | --- | --- | --- | --- |
| Forward Primer | | CAACATACAGTGACGCTTCACA | | Amplicon Size  105bp | PrimerBank ID  116875766c1 |
| Reverse Primer | | CACTATTGACGTTTCCCCACTC | |  |  |
| **Homo sapiens claudin 1 (CLDN1)** | | | | | |
| Forward Primer | | CCTCCTGGGAGTGATAGCAAT | | Amplicon Size  145 | PrimerBank ID  296785063c1 |
| Reverse Primer | | GGCAACTAAAATAGCCAGACCT | |  |  |
| **Homo sapiens glyceraldehyde-3-phosphate dehydrogenase (GAPDH)** | | | | | |
| Forward Primer | GGAGCGAGATCCCTCCAAAAT | | Amplicon Size  197 | | PrimerBank  ID378404907c1 |
| Reverse Primer | GGCTGTTGTCATACTTCTCATGG | |  |  |  |
